# Hyperthermic intraperitoneal chemotherapy with raltitrexed for peritoneal recurrence presenting with massive ascites following radical surgery for advanced gastric cancer: a case report

**DOI:** 10.3389/fonc.2026.1701376

**Published:** 2026-02-17

**Authors:** Wenjie Xie, Li Deng, Rongkun Mou, Kuan Liu

**Affiliations:** 1 Department of General Surgery, The Third Hospital of Mianyang, Sichuan Mental Health Center, Mianyang, Sichuan, China; 2 Department of pediatrics, The Third Hospital of Mianyang, Sichuan Mental Health Center, Mianyang, Sichuan, China

**Keywords:** case report, gastric cancer, hyperthermic intraperitoneal chemotherapy, peritoneal recurrence, raltitrexed

## Abstract

Peritoneal recurrence is the most common form of recurrence after radical gastrectomy for gastric cancer (GC) and is the main cause of death. The median survival period is 3–6 months, and the 5-year survival rate is almost zero. So far, the treatment methods recommended by various clinical guidelines for peritoneal metastasis (PM) of GC are all palliative systemic chemotherapy (SC). However, the therapeutic effect is not ideal. One possible reason is the existence of the blood-peritoneal barrier, which makes it difficult for drugs to reach the peritoneal metastatic foci fully. Based on this, surgeons have gradually attempted the intraperitoneal administration mode in clinical practice. Hyperthermic intraperitoneal chemotherapy (HIPEC) improves the anti-tumor efficacy through the synergistic effect of heat and chemotherapy drugs and has achieved good therapeutic effects in the treatment of PM of GC. Raltitrexed is mainly used for intravenous administration and intraperitoneal chemotherapy for advanced colorectal cancer. HIPEC with raltitrexed for the treatment of PM after radical gastrectomy for GC is extremely rare. This article introduces a case of advanced GC with peritoneal recurrence and a large amount of ascites 11 months after radical gastrectomy. After 5 cycles of HIPEC with raltitrexed, a favorable therapeutic effect was achieved. The patient experienced significant clinical improvement, characterized by the resolution of ascites, alleviation of symptoms, and prolonged survival, thereby creating an opportunity for subsequent comprehensive treatment. It is hoped that this case can provide a treatment option for patients with peritoneal recurrence after radical gastrectomy for GC.

## Introduction

Gastric cancer (GC) ranks among the most prevalent malignancies globally and is the fifth leading cause of cancer-related mortality (1). Its poor 5-year survival rate is primarily attributed to tumor progression and recurrence. Following radical resection, the peritoneum represents the most frequent site of metastasis in GC patients, particularly those with serosal infiltration or lymphatic involvement. Peritoneal metastasis (PM), occurring in 20%–30% of gastric cancer patients, arises predominantly from the exfoliation of free cancer cells within the abdominal cavity. The prognosis for PM is dismal, with a median overall survival of 3–6 months without treatment and no reported long-term survival (2, 3). Current clinical guidelines recommend only systemic palliative chemotherapy (pSC) and best supportive care as treatment options. However, outcomes with chemotherapy alone are suboptimal, largely due to the limited penetration of chemotherapeutic agents across the blood-peritoneal barrier to reach peritoneal implants (4).

Since the 1990s, the concepts of cytoreductive surgery (CRS) and hyperthermic intraperitoneal chemotherapy (HIPEC) have been developed to address the limitations of pSC. CRS involves the surgical removal of all macroscopically visible disease. HIPEC concurrently delivers heated, high-concentration chemotherapeutic agents directly into the peritoneal cavity. This approach sustains a thermotherapeutic effect and maximizes drug exposure to residual cancer cells, thereby enhancing antitumor efficacy. The combination of CRS and HIPEC is now established as the standard treatment for certain peritoneal surface malignancies (5, 6). Consequently, the integration of HIPEC with systemic chemotherapy is emerging as a potential strategy for both preventing and treating PM in various cancers. Raltitrexed is a specific thymidylate synthase inhibitor characterized by a prolonged plasma terminal half-life and demonstrated activity against gastrointestinal malignancies. This extended half-life positions raltitrexed as a promising candidate drug for intraperitoneal administration.

## Case presentation

**Figure 1:**
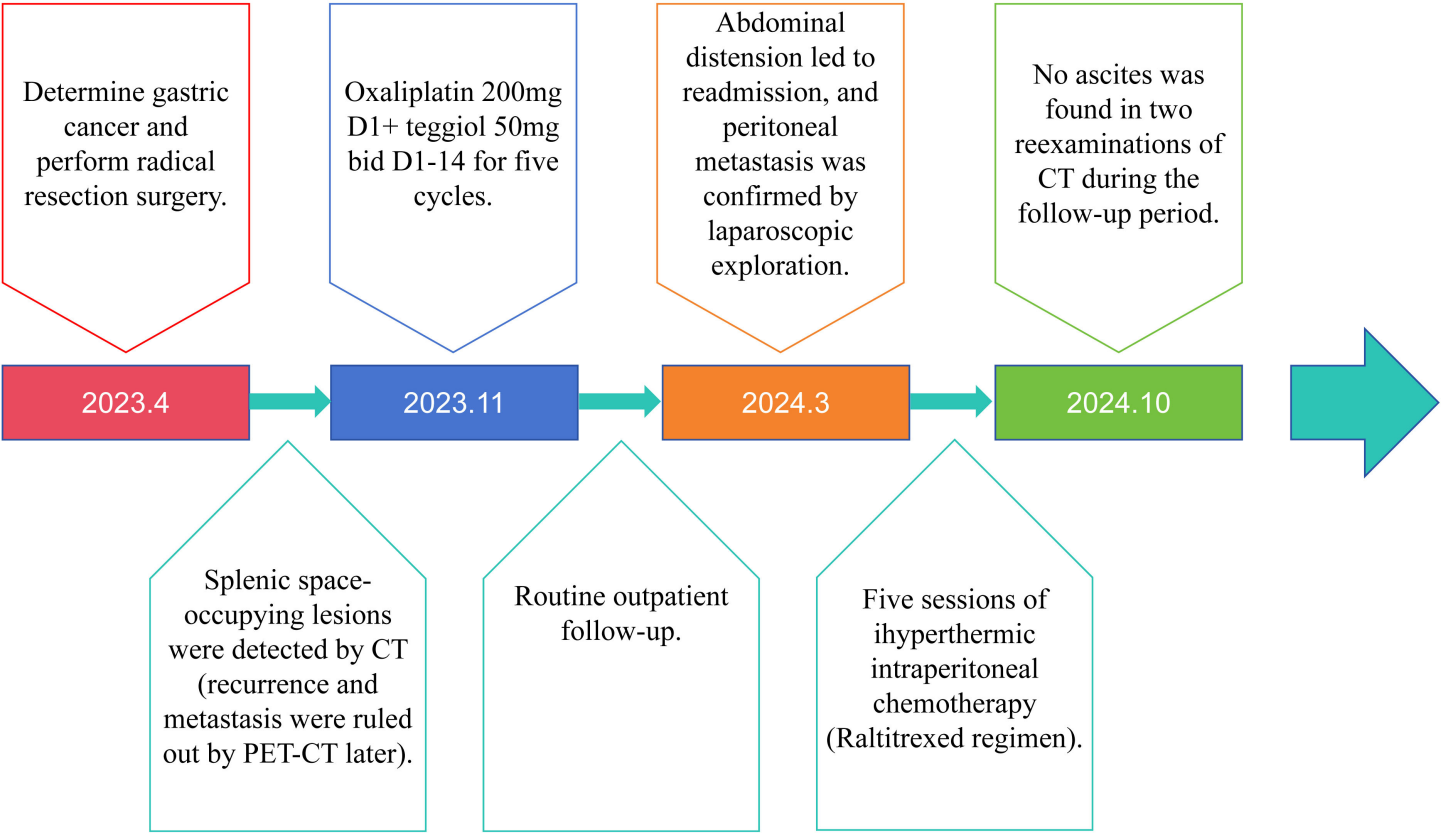
Timeline of patient clinical events.

The timeline of the 76-year-old male patient in this study is shown in **Figure 1**.

**Figure 2:**
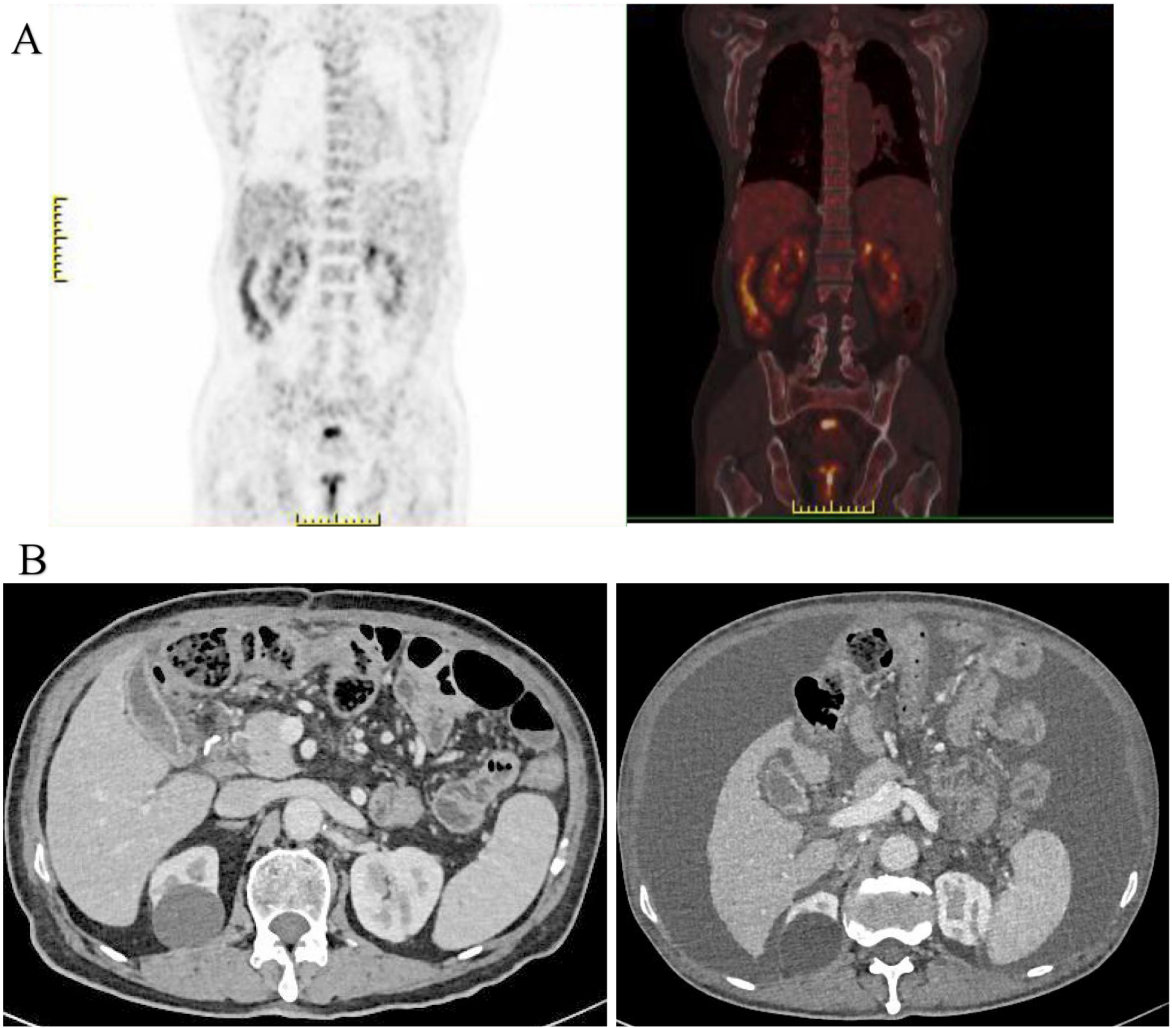
**(A)** PET-CT imaging demonstrated no findings suggestive of tumor recurrence; **(B)** Enhanced abdominal CT revealed a significant accumulation of fluid in the abdominal cavity compared to previous findings (August 2023).

On April 10, 2023, the patient was admitted to our hospital due to “abdominal fullness with dull pain and discomfort for 10 days”. Physical examination revealed mild upper abdominal tenderness only, with no other positive signs. Gastroscopy identified an ulcerated tumor in the gastric body. Pathological examination of the biopsy specimen confirmed poorly differentiated adenocarcinoma. Contrast-enhanced abdominal computed tomography (CT) demonstrated gastric carcinoma with regional lymphadenopathy. No significant abnormalities were detected on further auxiliary investigations. The initial diagnosis was gastric body adenocarcinoma (cT3N0M0, Stage II). Following a multidisciplinary team (MDT) discussion, surgical intervention was indicated. The patient underwent radical total gastrectomy under general anesthesia on April 13, 2023. Final histopathological staging revealed gastric body adenocarcinoma (pT4aN3bM0, Stage IIIC). The patient’s postoperative recovery was uneventful, and he was discharged. Adjuvant chemotherapy was subsequently initiated, consisting of six cycles administered on the following dates: May 29, 2023; June 28, 2023; July 27, 2023; August 31, 2023; October 14, 2023; and November 13, 2023. The regimen comprised: Oxaliplatin 200mg ivgtt D1+ S-1 50mg po bid D1-14. Notably, a surveillance CT scan performed during the fourth chemotherapy cycle (August 2023) revealed a splenic mass, suggestive of metastatic disease. However, a subsequent positron emission tomography-computed tomography (PET-CT) scan showed no evidence of tumor recurrence (**Figure 2A**).

Following regular outpatient surveillance, the patient was readmitted to the hospital due to “abdominal distension for 7 days” on March 12, 2024. Physical examination on admission showed a distended abdomen, frog-like abdomen, old surgical scars on the abdomen, soft and non-tender, and other abnormalities. Abnormal laboratory findings included: hemoglobin, 94g/L (normal 130-175g/L); Albumin, 30.9g/L (normal 40-55g/L); K, 2.77mmol/L (normal 3.5-5.5mmol/L); CA-125: 59.7U/ml (normal 0-30.2U/mL). Enhanced abdominal CT revealed a massive peritoneal fluid collection (**Figure 2B**). Due to the late stage of the tumor, the possibility of tumor recurrence was considered, and the exfoliative cytology of the tumor was performed twice; the results were negative. After the MDT discussion, laparoscopic exploration was recommended. The reasons included: First, after radical total gastrectomy, the stage was adenocarcinoma of the gastric body (pT4aN3bM0, III stage), and the recurrence rate was very high; Second, no malignant tumor cells were found in two exfoliative cytology examinations; Third, laparoscopic exploration can help to confirm whether it is recurrent gastric cancer and guide the follow-up strategy; Fourth, patients may benefit from HIPEC treatment of recurrent GC.

**Figure 3:**
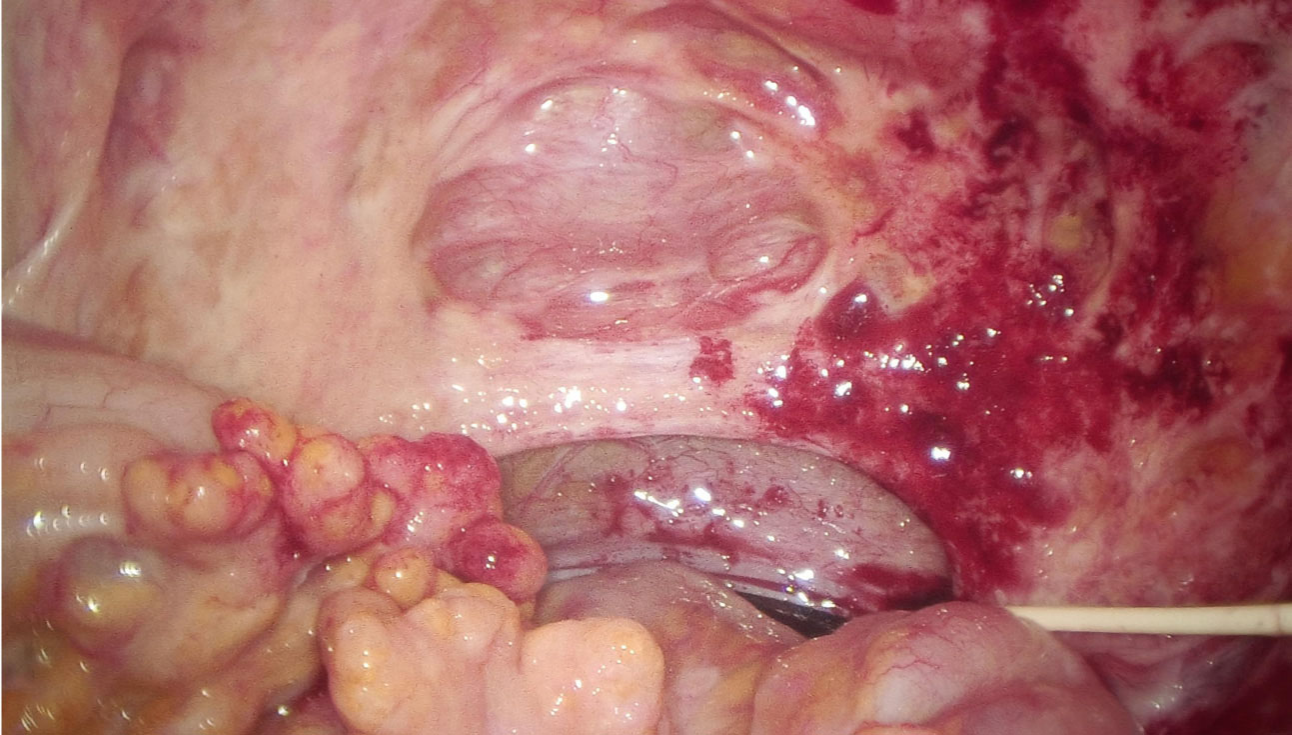
Laparoscopic exploration revealed recurrence of abdominal tumors.

Consequently, laparoscopic exploration was performed on March 21, 2024. During the operation, it was found that the small intestine and its mesentery, the colon and its mesentery, and the pelvic peritoneum had multiple yellow-white nodules with tough texture. Part of the wall peritoneum was red, and a small amount of blood oozed. A large volume of clear yellow ascites was present within the abdominal and pelvic cavities (**Figure 3**). The white matter hard tissue was taken from the abdominal cavity and the top of the pelvic cavity and sent for intraoperative frozen. And the final pathological examination suggested adenocarcinoma. An intraperitoneal perfusion catheter system was placed for subsequent HIPEC. Postoperative HIPEC was administered on March 25, 26, 28, 30, and April 3, 2024. The procedure of hyperthermic intraperitoneal chemotherapy (HIPEC) was performed using the BR-TRG Body Cavity Hyperthermic Perfusion Therapy System (Bright Medical). Specifically, 4 mg of raltitrexed was added to 4000 mL of normal saline. The solution was heated by the circulation unit of the hyperthermic perfusion device and then infused into the abdominal cavity through four abdominal drainage tubes connected to the therapeutic perfusion circuit. During the treatment, approximately 1500-3500 mL of perfusate was maintained in the peritoneal cavity, with a flow rate controlled between 400 and 600 mL/min. The perfusion was continuously carried out at a constant temperature of 43 °C for 60 minutes. During the perfusion procedure, the patient reported mild abdominal distension. Following treatment, the patient developed grade 1 myelosuppression (as graded by CTCAE (Common Terminology Criteria for Adverse Events) v5.0), representing a mild adverse event, which improved after symptomatic management. An abdominal ultrasound performed on day 5 post-treatment revealed no significant fluid collection, and the drainage tubes were subsequently removed prior to hospital discharge.

**Figure 4:**
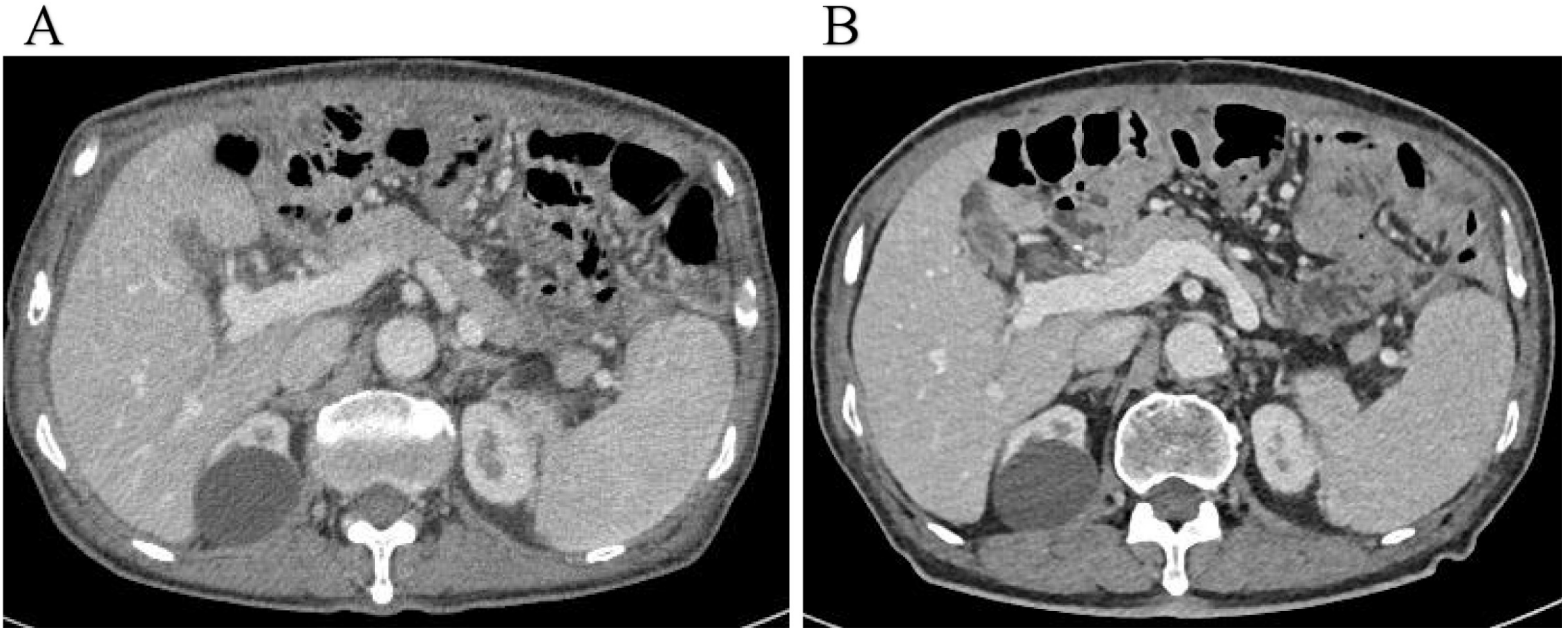
**(A, B)** Surveillance abdominal CT scans performed in May and October 2024 showed no recurrence of ascites.

Five days after the final HIPEC session, abdominal ultrasound revealed no residual ascites, and the perfusion catheters were removed before discharge. The patient was very satisfied with the treatment effect and would continue with systemic palliative chemotherapy in the oncology department subsequently. Surveillance abdominal CT scans performed in May and October 2024 showed no recurrence of ascites (**Figure 4**).

## Discussion

GC ranks as the fifth most common malignancy and leading cause of cancer-related mortality worldwide, with an overall 5-year survival rate of approximately 25%. Synchronous peritoneal metastases are present in up to 40% of patients at initial diagnosis (1, 4). Peritoneal recurrence following gastric cancer surgery carries a dismal prognosis, with a median survival not exceeding 6 months and a 5-year survival rate approaching zero (7). With ongoing research, initial recommendations for peritoneal dissemination of GC involved pSC or best supportive care. However, for selected patients, the latest NCCN guidelines suggest that CRS+HIPEC is also a viable option. A key advantage of CRS+HIPEC is its ability to overcome the peritoneal-plasma barrier, enabling effective drug delivery into the peritoneal cavity (8, 9). Current studies indicate that compared with pSC, CRS+HIPEC offers a significant survival advantage for patients with PM (10–12).

Research on treating advanced malignancies continues to advance. Evidence suggests that intraoperative lavage combined with perioperative intraperitoneal chemotherapy (IPC) effectively eliminates free cancer cells, with reported efficacy potentially 10 to 1000 times greater than intravenous chemotherapy (13). This regimen has been shown to significantly improve five-year survival rates. Furthermore, studies indicate a strong correlation between the plasma concentration of chemotherapeutic agents and their cytotoxic efficacy; specifically, an increase in intratumoral drug concentration at a critical time point can enable the elimination of a tenfold greater number of cancer cells (14).

While the efficacy of IPC is increasingly well-established, optimal agent selection remains contentious. Numerous drugs, including melphalan, 5-fluorouracil (5-FU), mitoxantrone, doxorubicin, and topotecan, have been administered intraperitoneally. In GC management, cisplatin, paclitaxel, 5-FU, and carboplatin are commonly utilized, with cisplatin being the most extensively studied agent based on comprehensive clinical data. Nevertheless, cisplatin’s substantial adverse effect profile poses significant clinical challenges. Moreover, prolonged use frequently induces resistance in most patients, leading to elevated recurrence rates. Compounding these issues, cisplatin’s relatively low molecular weight promotes rapid systemic absorption, potentially intensifying systemic toxicity (15, 16). Consequently, identifying optimal agents for IPC is paramount.

Peritoneal drug clearance is governed by both drug characteristics and peritoneal physiology. Generally, agents possessing higher molecular weights typically display lower lipophilicity, resulting in slower clearance from the peritoneal cavity. This pharmacokinetic profile extends local drug exposure, enhancing tumor tissue penetration and consequently augmenting cytotoxicity against malignant cells. Raltitrexed (molecular weight: 458), significantly larger than cisplatin or 5-FU, possesses an extended plasma half-life of 196 hours. This characteristic facilitates prolonged interaction with cancer cells without requiring sustained-release formulations. Additionally, raltitrexed is a specific, water-soluble inhibitor of thymidylate synthase. Its reduced folate metabolite undergoes active cellular transport via membrane carriers and is subsequently metabolized intracellularly to polyglutamate derivatives, which exhibit sustained intracellular activity. Collectively, these attributes—slower clearance, prolonged half-life, specific mechanism of action, and sustained intracellular activation—position raltitrexed as a highly promising IPC candidate. Emerging evidence suggests raltitrexed may offer advantages over 5-FU for IPC, supported by favorable safety profiles observed with its intraperitoneal administration (17). Raltitrexed also plays a significant role in other tumors. Studies have indicated that Raltitrexed can serve as a reliable option for chemotherapy in locally advanced or metastatic colorectal cancer, particularly for patients intolerant to 5-FU (18). Evidence also supports its reliability, efficacy, and safety in the intraperitoneal chemotherapy of colon cancer (19). Furthermore, Chen et al. demonstrated the efficacy and safety of Raltitrexed plus Oxaliplatin via hepatic arterial infusion chemotherapy combined with Apatinib as a second-line treatment in patients with advanced hepatocellular carcinoma and extrahepatic metastases (20). Similarly, Lu et al. confirmed that the aforementioned regimen could improve survival and safety outcomes in elderly patients with unresectable hepatocellular carcinoma (21).

While HIPEC with raltitrexed is primarily investigated for preventing PM post-radical gastrectomy, its application for treating established peritoneal recurrence in advanced GC remains unexplored. In this case, the patient received five HIPEC with raltitrexed cycles following peritoneal recurrence. Treatment resulted in significant clinical improvement: complete resolution of ascites, increased appetite, improved nutritional indices, and no ascites recurrence during 6 months of follow-up. This outcome substantially exceeds the expected median survival of 3–6 months for untreated PM, suggesting HIPEC with raltitrexed may offer meaningful disease control, symptom palliation, and survival prolongation in this setting. Study Limitations: This report describes a single patient experience. Consequently, the findings may lack broad generalizability, and validation through larger prospective studies is warranted.

## Conclusion

Treatment options for postoperative peritoneal recurrence of advanced GC are limited. In this study, we present the case of a 76-year-old male patient with locally advanced GC that progressed to peritoneal carcinomatosis, who underwent five sessions of HIPEC with raltitrexed, which resulted in the complete disappearance of ascites. Notably, no recurrence of ascites was observed during the 6-month follow-up period, contributing to prolonged survival and improved quality of life. In conclusion, HIPEC with raltitrexed represents a viable therapeutic option for managing peritoneal recurrence in advanced GC and may achieve favorable clinical outcomes.

## Data Availability

The raw data supporting the conclusions of this article will be made available by the authors, without undue reservation.
